# Interrater Reliability of Motion Palpation in the Thoracic Spine

**DOI:** 10.1155/2015/815407

**Published:** 2015-06-11

**Authors:** Bruce F. Walker, Shane L. Koppenhaver, Norman J. Stomski, Jeffrey J. Hebert

**Affiliations:** ^1^School of Health Professions, Murdoch University, Perth, WA 6150, Australia; ^2^US Army-Baylor University Doctoral Program in Physical Therapy, San Antonio, TX 78234-6100, USA; ^3^School of Psychology and Exercise Science, Murdoch University, Perth, WA 6150, Australia

## Abstract

*Introduction*. Manual therapists commonly use assessments of intervertebral motion to determine the need for spinal manipulation, but the reliability of these procedures demonstrates conflicting results. The objectives of this study were to investigate the interrater reliability of thoracic spine motion palpation for perceived joint restriction and pain.* Methods*. Twenty-five participants between the ages of 18 and 70, with or without mid-back pain, were enrolled. Two raters motion palpated marked T5–T12 levels using two methods (standardised and pragmatic) and noted any restricted or painful segments. We calculated agreement between two raters by generating raw agreement percentages and Kappa coefficients with 95% confidence intervals.* Results*. There was poor to low level of agreement between the raters for both joint stiffness and pain localization using both pragmatic and standardized approaches. The results did not improve significantly when we conducted a post hoc analysis where three spinal levels were collapsed as one and right and left sides were also combined.* Conclusions*. The results for interrater reliability were poor for motion restriction and pain. These findings may have unfavourable implications for all manual therapists who use motion palpation to select patients appropriate for spinal manipulation.

## 1. Introduction

Assessment of intervertebral motion is considered a fundamental component of the clinical examination by many manual therapy providers. In addition to a clinical history such assessment is often used to identify primary areas of joint restriction in patients with spinal pain. Additionally, assessment of intervertebral motion is commonly performed in patients with primary complaints of other regions (e.g., shoulder, hip pain) in order to identify changes in movement that may be related to such complaints [1–3]. Restricted intervertebral motion has classically been considered a key indicator of spinal dysfunction by many chiropractors [4]. Moreover, a recent survey among Australian physiotherapists found that the great majority (98%) of manual therapy physiotherapists use manual assessments of spinal motion during their exam and base treatment decisions on their findings [5].

Establishing reliability of an examination procedure is generally considered a prerequisite for its validity and clinical utility [6, 7]. In the context of this study reliability is defined as the degree of stability exhibited when a measurement is repeated under identical conditions [8]. The reliability of intervertebral motion assessment has been extensively studied and systematically reviewed for the cervical and lumbar regions [9–12]. Although reliability estimates of intervertebral motion assessment vary widely, systematic reviews report substantial methodological shortcomings with the majority of these studies [10–12]. The latest systematic review focusing solely on reliability studies of intervertebral motion assessment of the lumbar and cervical spine [12] found that only four out of 19 included studies were performed in participants with musculoskeletal complaints and that only three of the 19 studies included examiners that were blinded to each other's assessments. Although inconclusive due to these limitations, the majority of studies, especially those of higher quality [9, 12], report poor reliability, often no better than chance [10–12].

Few original studies have specifically investigated intervertebral motion assessment of the thoracic spine and these have provided inconsistent evidence regarding the reliability of motion palpation [13–16]. However, a growing body of research [17] and clinical commentary [18] supports the importance of including thoracic spine function in the evaluation of people with neck and shoulder complaints. Specifically, recent studies suggest that the use of spinal manipulation to the thoracic spine may be beneficial to patients with neck pain [19–23] and shoulder impingement syndrome [24–26]. Since manual therapy practitioners commonly use assessments of intervertebral motion to determine what specific segment or region of the thoracic spine to apply spinal manipulation, there remains a need to investigate the reliability of such procedures. Accordingly, we investigated the interrater reliability of assessments of thoracic spine intervertebral motion and pain in a sample that included participants with both primary complaints of thoracic pain and those without primary complaints of thoracic pain. Additionally, we evaluated reliability of assessments both when experienced examiners applied pragmatic and highly standardized approaches.

## 2. Methods

### 2.1. Participants

Twenty-five volunteers between the ages of 18 and 70, with or without mid-back pain, were recruited from a university campus. Participants were excluded if they had fibromyalgia, rheumatoid arthritis, ankylosing spondylitis, or other inflammatory spinal disease or could not tolerate the physical examination. The study protocol was approved by the Murdoch University Human Research Ethics Committee, and all participants provided written informed consent prior to enrolment.

### 2.2. Examiners

Two experienced chiropractors (raters A and B) participated as examiners for the study. Both had current academic appointments, came from a clinical background, and had taught motion palpation in an academic setting. Each examiner had their own style of motion palpation but was also familiar with other standardised techniques. We assessed the reliability of using both their own pragmatic approach and a standardised technique of intervertebral motion assessment.

### 2.3. Examination Procedures

Participants were examined by both raters twice, once with their own pragmatic motion palpation method and once with a standardised method of motion palpation as described by Bergman and Peterson [1]. During the pragmatic procedures, examiners used the method they would normally use to assess intervertebral motion in a clinical setting. The standardized procedure consisted of assessing passive physiologic motion of flexion, extension, bilateral side bending, and bilateral rotation, followed by segmental intervertebral mobility testing. Each rater was blinded to whether or not each participant had back pain and to the findings of the other rater. In addition the order in which participants were examined by each rater was changed on the second round to minimise any memory of findings. Participants were seated on a fixed examination table separated from each other so that the raters could not hear each other's conversation. The examiner was free to move into a position comfortable for them to examine the participant.

#### 2.3.1. Nonstandardized Assessment

**Figure 1:**
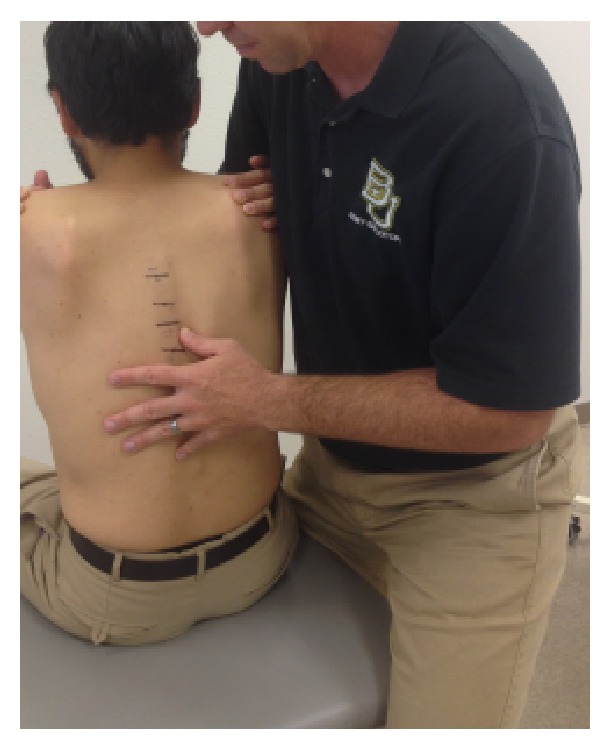
Motion palpation setup.

Participants were seated on an examination table separated from each other so that the raters could not hear each other's conversation. A third experienced examiner labelled the spinous processes of each participant from the T5 to T12 spinal levels in order to eliminate potential error in identifying the correct spinal level. The raters conducted their motion palpation assessment (Figure 1) and then moved on to the next participant. Rater A was the first to commence using his own usual approach. Then rater B followed and examined the same 25 participants.

#### 2.3.2. Standardised Assessment

Following the nonstandardised assessment, we conducted a 30-minute training session, followed by a practice series involving 4 student volunteers to demonstrate and standardize the motion palpation approach [1]. Once both raters were satisfied that they were competent in the new procedure, the testing session was repeated using this standardised motion palpation method.

First, the participants were seated on a chair with arms by their sides, the mid back skin exposed, and the marks on the spinal processes visible. Raters were asked to motion palpate the marked T5–T12 levels and note any restricted or painful segments and whether it was on the right or the left. To simulate clinical practice the rater was free to ask the participants if “it hurts” during their segmental examination and pain was recorded if they received an affirmative answer. A restricted segment was defined as having a loss of joint play [27] which was perceived as hypomobile such that the examiner would consider it appropriate for manipulation.

### 2.4. Data Analysis

Data management and analyses were performed using the Statistical Package for the Social Sciences version 17 software (SPSS, Chicago, IL). Descriptive statistics, including estimates of central tendency and variability, were calculated to describe the sample of participants.

Interrater reliability of motion assessment was estimated separately for judgments of motion restriction (yes/no) and pain with motion (yes/no), each during the nonstandardized (pragmatic) approach and the standardized approach. We calculated agreement between two raters by generating raw agreement percentages and Kappa coefficients with 95% confidence intervals. Kappa statistics represent the proportion of agreement greater than that expected by chance and are traditionally interpreted as representing excellent agreement above 0.80, substantial agreement between 0.61 and 0.80, moderate agreement between 0.41 and 0.60, fair agreement between 0.21 and 0.40, and slight agreement between 0.00 and 0.20 [28]. Negative Kappa values indicate agreement less than chance. When interpreting Kappa coefficients, however, it is important to understand that both bias and prevalence have potential to influence the agreement estimates. Bias occurs when there is disagreement in the proportion of yes and no judgments between each rater. As bias increases, chance agreement decreases, resulting in inflation of the Kappa coefficient. With large differences in the prevalence of yes versus no judgments, there is increased chance agreement, which lowers the Kappa coefficient. To account for these potential sources of error and enhance the interpretation of the Kappa statistics, we additionally calculated indices of prevalence and bias as well as prevalence-adjusted and bias-adjusted Kappa coefficients (PABAK) [29, 30].

## 3. Results

The demographic and clinical characteristics of the 25 participants are listed in Table 1. Five participants had mid-back pain on the day of assessment and 18 had previous mid-back pain. Interexaminer agreement estimates are listed in Table 2. Kappa statistics for judgments of segmental restriction ranged between −0.27 and 0.36 depending on the spinal level. Point estimates for level of agreement using the nonstandardized (pragmatic) approach were generally worse than would be expected by chance alone. Point estimates for level of agreement using the standardized approach were generally better than would be expected by chance alone, although the 95% CI of all but one estimate (T6–8) included zero.

Findings regarding agreement of segmental pain were similar. Kappa statistics ranged between −0.38 and 0.32 depending on the spinal level. Point estimates for level of agreement using the nonstandardized (pragmatic) approach were generally worse than would be expected by chance alone. Point estimates for level of agreement using the standardized approach were generally better than would be expected by chance, although the 95% CI of all estimates included zero.

## 4. Discussion

This was the first study to evaluate the interrater reliability of motion palpation of the thoracic spine both when experienced examiners were free to use their own nonstandardized assessment methodology and when assessments were highly standardized. Regardless of the standardization condition, reliability for pain and motion restriction results was poor and in many cases was not better than chance.

It can be argued that it may be more challenging to determine intersegmental motion of the thoracic spine using motion palpation compared to the cervical and lumbar regions due to the inherent reduced thoracic intersegmental movement due to the attachment of the rib cage. However, this is speculative and there is no evidence to substantiate this notion. Although assessment of motion palpation of the lumbar and cervical spine has been exhaustively studied, the few studies that have investigated intervertebral motion assessment of the thoracic spine have reported conflicting results [13–16]. Using methodology very similar to that of the current study, Christensen et al. [14] found interexaminer reliability of motion palpation of the thoracic spine that was in-line with our findings of essentially no better than chance with Kappa (95% CI) = 0.00 (−0.52,0.52). Reliability was improved slightly when they allowed for “expanded agreement” in which they considered examiners to agree if they were within one spinal level of each other, but still statistically not different than chance (Kappa (95% CI) = 0.22 (−0.29,0.73)). Also using very similar methodology of the current study, Brismée et al. [13] found slightly better reliability (*k* = 0.27 to 0.65) depending on the examining pair. Lastly, using a different methodology, Cooperstein et al. [15] assessed prone participants for the most restricted thoracic vertebral segment and then measured it as distance from S1. Interexaminer agreement overall was poor [ICC (95% CI)  =  0.31 (0.04,0.54)] except when examiners reported being “very confident” about their results [ICC (95% CI)  =  0.83 (0.63,0.93)].

The lack of reliability of pain assessment in the current study was unanticipated. Previous studies in the lumbar and cervical spine have generally found that assessments of pain provocation during intervertebral motion assessments are much more reliable than assessments of limited mobility [10, 11]. In the current study, assessments of segmental pain were not consistently more reliable than assessments of segmental motion restriction. In the single previous study to evaluate reliability of segmental pain assessment in the thoracic spine, Christensen et al. [14] found interexaminer reliability of zygapophyseal joint tenderness to be low to moderate (Kappa  =  0.38) when using strict agreement criteria and good (Kappa  =  0.67–0.70) when they considered examiners to agree if they were within one spinal level of each other. Although the reason for our differing findings is unknown, they might simply be due to methodological differences between the current and previous studies.

Establishing reliability of an examination procedure is generally considered a prerequisite for its validity and clinical utility [6, 7]. If this is the case, then assessment procedures demonstrated to be unreliable should not be useful clinically. However, although no studies to our knowledge have evaluated the validity of thoracic motion palpation, numerous studies have evaluated validity of motion palpation in the cervical and lumbar spine with mixed results [31–34]. In a recent study in the lumbar spine, Koppenhaver et al. [34] found essentially no correlation between manual assessment of segmental stiffness and a criterion measure using spinal indentation. Conversely, Humphreys et al. [33] found that manual practitioners were able to identify the level of restricted cervical motion in individuals with congenitally blocked vertebrae. However, the question arises whether congenitally blocked vertebrae would have a similar end-feel to that of a notionally restricted segment. Moreover, some research in the lumbar spine suggests that assessment of intervertebral motion is helpful in making treatment decisions. Specifically Fritz et al. [32] performed secondary analyses of data from a randomized controlled trial and found that patients who were judged as “hypermobile” did best with lumbar stabilization exercises and those that were judged as “hypomobile” did best with spinal manipulation treatment. Such findings suggest that manual assessments of spinal mobility may be sufficiently reliable to be a useful component of the clinical examination. Unlike the current study, however, spinal mobility was generally classified across the entire lumbar spine rather than segment by segment.

Previous research has reported improved reliability when more broadly categorizing a region of the spine than when judging mobility at a specific spinal level [10] or when considering agreement to occur when within one spinal level [14]. To evaluate the impact of this in the current study, we performed additional post hoc analysis in which we collapsed the data for levels in the following way: right and left sides for each vertebral level were collapsed and then the data reanalyzed and similarly we further collapsed vertebral level to 3 contiguous vertebrae and then also reanalyzed the data. The rationale for collapsing the side of involvement and level of involvement was to allow for the contingency that the rater was palpating some functional loss of joint play at a specific level which may have been caused by similar stiffness from the contralateral side or from a level above or below. This further analysis did not show an improvement in agreement between raters.

*Study Limitations*. This study had several limitations. The sample size was under the number contained in recommendations [7], which may have resulted in an underestimation of the Kappa values. Nonetheless, all Kappa values reported in this study were consistently low and the confidence intervals commonly crossed zero, which suggests that the results are unlikely to appreciably change with an increased sample size. The examiner training was brief and appeared to be adequate, but more extensive training may have resulted in a higher level of agreement. Our outcome measures were perceived stiffness and subjective pain/tenderness, and whether these measures remained constant in individuals between examinations is unclear. Finally, if a rater put their finger on a marked line then the level was left to the interpretation of the recorder; however, as the results did not change when levels were collapsed this is unlikely to have provided any bias.

## 5. Conclusion

Regardless of the degree of standardization, interrater reliability of motion palpation of the thoracic spine for identifying pain and motion restriction performed by experienced examiners was poor and often not better than chance. These findings question the continued use of motion palpation as part of the clinical assessment as an isolated tool to detect loss of intersegmental joint play.

## Figures and Tables

**Table 1:** Demographic and clinical characteristics of participants, *N* = 25.

Characteristic	Value
Height (cm)	175 (12.2)
Weight (kgs)	71 (11.3)
BMI (kg/cm^2^)	22.8 (3.2)
% female	40%
% with current mid-back pain	20%
% with previous mid-back pain	70%
Number of previous episodes	2.7 (1.4)
Time since last episode (months)	3.9 (1.8)
Intensity of last episode (0–10)	3.8 (2.3)

Note: values are mean (SD) unless otherwise indicated.

**Table 2:** Interexaminer agreement.

Spinal level	Kappa	95% CI	% agreement	Prevalence index	Bias index	PABAK
Nonstandardized approach to assessment of segmental restriction						
T5–7	0.12	−0.02, 0.27	44	−0.20	0.56	−0.12
T6–8	−0.14	−0.48, 0.18	40	−0.16	0.28	−0.20
T7–9	−0.05	−0.44, 0.35	48	−0.08	0.04	−0.04
T8–10	−0.27	−0.63, 0.10	36	0.12	0.16	−0.28
T9–11	−0.16	−0.45, 0.12	32	0.16	0.44	−0.36
T10–12	0.19	0.00, 0.39	52	0.20	0.48	0.04

Standardized approach to assessment of segmental restriction						
T5–7	0.36	0.00, 0.72	68	0.04	−0.08	0.36
T6–8	0.19	0.20, 0.57	60	−0.12	0.00	0.20
T7–9	−0.14	−0.48, 0.19	44	−0.28	0.24	−0.12
T8–10	0.09	−0.14, 0.31	44	−0.04	0.48	−0.12
T9–11	0.09	−0.15, 0.32	48	0.24	0.44	−0.04
T10–12	0.12	−0.10, 0.34	64	0.56	0.36	0.28

Nonstandardized approach to assessment of segmental pain						
T5–7	−0.25	−0.57, 0.07	44	0.36	0.16	−0.12
T6–8	−0.17	−0.55, 0.21	44	0.20	0.00	−0.12
T7–9	0.10	−0.25, 0.46	56	0.24	−0.20	0.12
T8–10	−0.38	−0.58, −0.18	44	0.44	−0.08	−0.12
T9–11	−0.15	−0.48, 0.17	52	0.44	0.16	0.04
T10–12	0.12	−0.17, 0.41	60	0.44	0.32	0.20

Standardized approach to assessment of segmental pain						
T5–7	0.29	−0.11, 0.68	72	0.48	−0.12	0.44
T6–8	0.32	−0.03, 0.68	68	0.28	−0.16	0.36
T7–9	0.27	−0.10, 0.64	64	0.16	0.12	0.28
T8–10	0.22	−0.08, 0.51	60	0.28	0.32	0.20
T9–11	0.09	−0.08, 0.25	56	0.48	0.44	0.12
T10–12	0.00	−0.26, 0.26	68	0.18	−0.18	0.00

N/A = not applicable, when at least one variable in each 2-way table upon which measures of association are computed is a constant.
